# *Acanthamoeba* Brain Abscess Confirmed by Molecular Identification

**DOI:** 10.4269/ajtmh.16-0375

**Published:** 2017-08-02

**Authors:** Sakda Wara-Asawapati, Pewpan M. Intapan, Verajit Chotmongkol

**Affiliations:** 1 Department of Pathology, Khon Kaen University, Khon Kaen, Thailand;; 2 Department of Parasitology and Research and Diagnostic Center for Emerging Infectious Diseases, Khon Kaen University, Khon Kaen, Thailand;; 3 Faculty of Medicine, Department of Medicine, Khon Kaen University, Khon Kaen, Thailand

**Figure 1.**
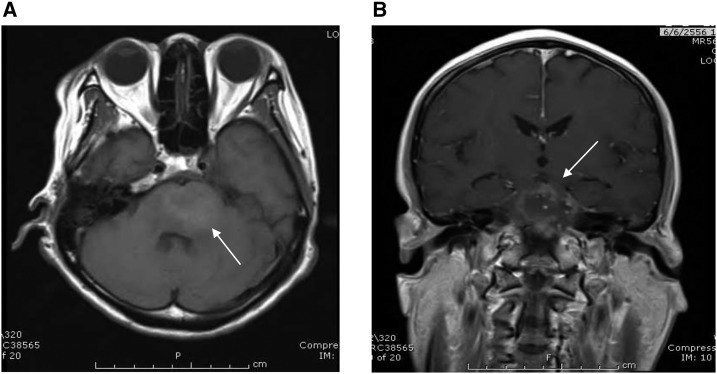
Magnetic resonance imaging of brain showed a large heterogeneous lesion and perilesional edema on the T1-weighted image (**A**) with rim enhancement (**B**) at pons.

**Figure 2.**
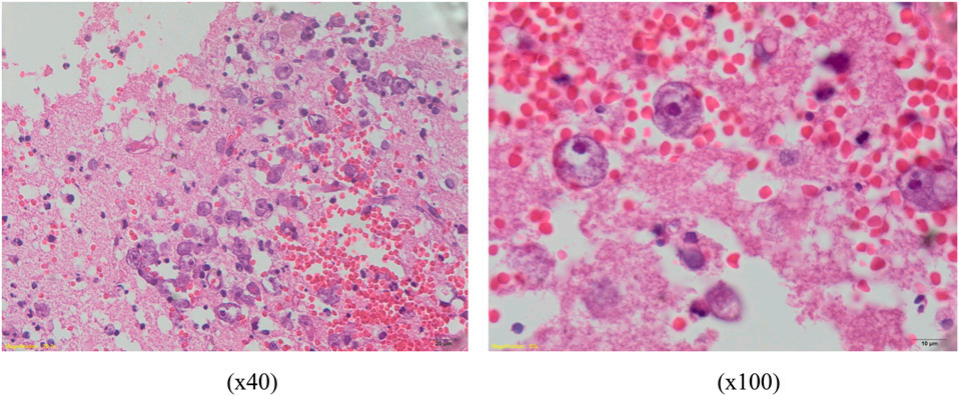
Hematoxylin and eosin–stained sections from paraffin blocks reveal many round-shape amoebae trophozoites with large karyosome and a halo-like appearance of the nucleus. This figure appears in color at www.ajtmh.org.

Clinical manifestations of infection of the brain caused by amoeba are divided into two types: primary amebic meningoencephalitis caused by *Naegleria fowleri* and focal brain lesion caused by *Entamoeba histolytica*, *Acanthamoeba* species, and *Balamuthia mandrillaris*. Early definite diagnosis and appropriate treatment are necessary for a good clinical outcome.^1,2^ A 58-year-old farmer woman, lived in rural area of the northeastern Thailand, presented with fever, alteration of consciousness, and progressive right hemiparesis for 10 days. She had a history of pulmonary tuberculosis and had undergone a complete course of treatment 2 years ago. Mixed connective tissue disease was also diagnosed 1 year ago due to history of Raynaud’s phenomenon, mild myositis, and positive high antinuclear antibody (ANA) titer (1:5,120); speckle type. She was treated with 10 mg of prednisolone for 2 months and then lost to follow-up. On physical examination, her body temperature was 38.2°C. She was in a state of stupor with right hemiparesis grade 0/5. No skin lesion was detected. Chest X-ray interpretation was normal. Magnetic resonance imaging of the brain revealed a 2.6 × 3.3 cm heterogeneous enhancing lesion, with rim enhancement and perilesional edema at pons (Figure 1). Other smaller lesions were found at the right cerebellar hemisphere, right occipital lobe, and right superior frontal gyrus. Craniotomy of the right frontal lobe revealed necrotic tissue. An excisional biopsy was conducted. Microscopic examination of the brain tissue showed acute inflammatory cell infiltration and many round-shaped protozoa. Antibody titer for *E. histolytica* in the serum was 1:512. Stool examination did not reveal the presence of any parasites. Abdominal sonography revealed a normal liver. The patient was treated with intravenous metronidazole 500 mg every 6 hours without improvement and finally died of the severe brain lesion. Reevaluation of histopathologic study revealed rounded amebic trophozoite with large karyosome and a halo-like appearance to the nucleus. (Figure 2). Molecular identification using *Acanthamoeba* genus-specific primers was positive and a 180-bp amplified product was found.^3^ No amplified product was found when the extracted DNA was done with specific primers for *E. histolytica*, *Naegleria*, and *Balamuthia*.^3–5^ The present results suggested a possible diagnosis of *Acanthamoeba* brain abscess.
